# The everyday life situation of caregivers to family members who have had a stroke and received the rehabilitation intervention F@ce in Uganda

**DOI:** 10.1186/s13690-021-00618-z

**Published:** 2021-06-15

**Authors:** Gunilla Margareta Eriksson, Julius Tunga Kamwesiga, Susanne Guidetti

**Affiliations:** 1 Division of Occupational Therapy, Karolinska Institutet, Stockholm, Sweden; 2 Rehabilitation Medicine, Uppsala University, Uppsala, Sweden; 3 Uganda Allied Health Examinations Board, Kampala, Uganda

**Keywords:** Rehabilitation, Telerehabilitation, Cell-phone, Africa, Caregiver burden, Life satisfaction, Counseling, Occupational therapy

## Abstract

**Background:**

Stroke is increasing in Africa and consequences such as limitations in the performance of activities in everyday life persist a long time. A family member might need to care for and assist the person who has had a stroke. The life situation of these caregivers thereby changes, which could lead to increased workload and new responsibilities in caring for which they lack but request knowledge. During the F@ce rehabilitation program, the caregivers received counseling, which is uncommon in the African context. The aim of the study was twofold; (1) to investigate the perceived caregiver burden and life satisfaction and, (2) to explore and describe the life situation for caregivers to persons that have had a stroke and received the mobile phone supported rehabilitation F@ce in urban areas in Uganda.

**Method:**

A mixed method design was used. Twelve caregivers took part in a semi-structured interview regarding their everyday life situation and responded to questionnaires on caregiver burden and life satisfaction. Latent qualitative content analysis was used to analyse the interviews.

**Results:**

Five categories were identified in the caregivers’ experiences of their life situation: *Feels obligated but is just a natural commitment; a tightly scheduled everyday life; being the supporting relative; the caregivers´ approach as rehabilitators; and being supported by the rehabilitation intervention.* The caregivers rated relatively high on the Caregiver Burden Scale and two thirds of the sample rated their satisfaction with life as a whole as dissatisfying. Further ratings on the Life Satisfaction checklist revealed that the financial, vocational, leisure and family situations were dissatisfying.

**Conclusions:**

Even if it was viewed as a natural commitment to be a caregiver when a family member had had a stroke, the life situation changed substantially for those who took on the caregiving role. Caregiving responsibilities were challenging as well as a heavy workload and a strained financial situation as many were giving up on jobs. The participants felt burdened and rated a low life satisfaction. The F@ce intervention was, however, expressed as valued and involved support and advice in their caregiving situation as well as information on stroke which relieved stress among them.

## Background

On a yearly basis, fifteen million people in the world suffer from stroke and of these, five million live with remaining impairment and activity limitations [1]. Stroke is increasing, particularly in Africa, afflicting young and middle-aged people [2, 3]. The onset of a stroke appears suddenly and may lead to motor and cognitive impairments as well as limitations in the everyday life activities, such as decreased ability to perform self-maintenance and household chores [4]. These consequences of stroke might persist for a long time [4] and were found to be one cause of chronic disease in Uganda [5]. A study on the impact of stroke in a chronic sample from Uganda found that strength, hand function, participation in activities as well as performing activities of daily living (ADL) such as self-care and performing household activities were the most impacted [6]. This implies that the consequences of a stroke can also affect everyday life negatively for the families that need to provide assistance. That is especially true in the African context where family members are those providing most of the care at home due to lack of healthcare and rehabilitation resources [2]. The caregivers in the family might have no previous experience of caring for someone who has had a stroke and may have to learn what this new role and responsibility involve and the change it brings about for their life situation [7, 8]. Furthermore, providing care is mostly a female responsibility according to African tradition, which adds to their general responsibility for the families’ overall health, food, security and child welfare [9, 10].

Quality of life for the caregiver as well as caregiver burden has been studied globally [11–14] by use of assessment instruments. Caregivers in low-income countries were found to rate the caregiver burden higher than in high-income countries [15] mostly due to a rough economic situation and poor support from healthcare. In the acute phase, caregivers perceived and rated severe strain [16] but the overall burden decreases over time [13, 16]. Furthermore, the portion of carers that perceive high burden also declined in the long term [13]. Quality of life (QoL) in a Nigerian sample was found to be lowest for those caregivers that had provided care for more than a year as well as for caregivers that were old and lacked formal education and employment [17]. Also female gender, older age and having a close relationship to the person having had stroke were related to lower QoL [9].

Caregivers to persons with stroke have described their experiences of being burdened and how the caregiving negatively affected their daily routines and their possibilities to participate in activities outside home, such as work and religious activities [10, 18]. Namale et al. [10] interviewed caregivers, mostly women, who had taken care of a family member with a stroke for at least four months, at discharge or shortly after discharge from an acute setting in Uganda. They described challenging experiences with difficult life changes, sudden new responsibilities, increased workload at home and changed life expectations. The women described feelings of being overwhelmed, stressed, tired and exhausted. These caregivers did not receive information on stroke or support and advice in their caring situation by healthcare personnel, which has also previously been emphasized as important [7, 19]. Research on caregivers’ experiences of everyday life while taking part in rehabilitation intervention for people with a stroke is lacking and is the focus of this study.

Provision of rehabilitation interventions after stroke is scarce in Sub-Saharan Africa, including Uganda, due to a poor health support system and infrastructure, as well as too few rehabilitation professionals and a poor socio-economic situation for the people. Information and support to caregivers are very limited when taking over the responsibility of caring for a person and dealing with his/her consequences of stroke while continuing with everyday life work without any previous knowledge and experience [10]. In the rehabilitation program F@ce, the families were provided information on stroke and advice on how to deal with consequences of stroke and how to assist in training. This eight-week program entailed goal setting, problem-solving strategies, daily SMS-reminders of the set goals as well as follow-ups on the training/activities performed. The rehabilitation program F@ce is described more in detail elsewhere [20]. There is a lack of knowledge on caregiver burden and satisfaction with life as well as how the life situation is experienced for caregivers that had access to counseling from rehabilitation personnel during a rehabilitation intervention for people who have had stroke. Therefore the aim of the study was to investigate the perceived caregiver burden and life satisfaction, and to explore and describe the life situation of caregivers to persons that have had a stroke and received the mobile phone supported rehabilitation F@ce in urban areas in Uganda.

## Method

### Design

A cross-sectional mixed method design was applied [21] using semi-structured qualitative interviews to collect the experiences of the family situation and established assessment instruments to describe life satisfaction and caregiver burden among relatives. These different types of data complemented each other and were chosen to reach a deeper understanding of the families’ life situations after a family member have had a stroke.

### Study context

The study was conducted in Kampala, the capital city of Uganda, and its surroundings. The participants were identified among the family members that belonged to a family where one person had had a stroke and was recruited to take part in the client-centered mobile-phone supported rehabilitation intervention F@ce [20]. The F@ce intervention aimed at increasing functioning in daily activities for persons living with the consequences of a stroke and participation in everyday life for the whole family. The persons with stroke and the caregiver met an occupational therapist (OT) once, who assessed the consequences of the stroke, provided information on stroke and rehabilitation interventions. Further, the OT collaborated with the family and formulated goals for daily activities the person wanted and needed to do as well as strategies for the daily training.

Each morning throughout the eight week-intervention, the person received SMS reminders on his/her phone about the goals and SMSs in the evening, which the person, alone or together with the caregiver, rated from 1 to 5 based on how well the activities chosen to be goals were performed. The OT called twice a week to discuss and give advice about the training. These calls were addressed to the person with stroke as well as to the caregiver.

### Participants

The participants were recruited when the persons with stroke started their participation in the F@ce intervention study. All family members who were relatives of persons with stroke were approached and informed about the study, both orally and in writing, and consented in writing to participate.

Twelve participants were included and were caregivers to persons that had participated in the F@ce intervention group. One of the caregivers who had family members taking part in the F@ce intervention (*n* = 13) could not be reached at the time of the interview. In addition to the F@ce intervention, six persons with stroke also received physiotherapy at a rehabilitation unit or in their own home. The mean age in the group was 37 years (information was missing for two participants) (range 20–50 years). Their educational level varied from primary school (*n* = 3), secondary school (*n* = 3), college (*n* = 1) and university level (*n* = 3) (information was missing for two participants). Of the 12 participants, nine were daughters, one was a son, one was a father and one was a niece. They all cared for their family members and four of the participants had quit their former job to be a caregiver, while two of them had started to work temporarily to manage the new situation with caring. A maid was hired for one of the persons that had had stroke. Seven of the participants did not work at all while the remaining participants managed to combine work or studies with caring for their loved one. Five of the participants were married and had children, four were not married but had children and, of those, one of the participant’s children was left to live on their own while the mother was caregiver to her aunt. Four other participants had also moved with or without their families to be caregivers. One of the participants was single and lived with the parent they cared for.

### Data collection

Data collection was performed at one point in time, between 6 and 24 months after onset of the family members’ stroke and after completing the F@ce intervention. Established assessments were used; these are commonly used in high-income countries, and developed in the Western world, to assess caregiver burden and life satisfaction among relatives to persons with stroke. The procedure in the data collection was to conduct the semi-structured interview first and thereafter pose the questions in the two assessments in a structured interview to clarify the alternatives, thereby making it easier for the participants to respond.

### Semi-structured interviews

A semi-structured interview guide including open-ended follow-up questions was developed to collect the participants’ experiences of the family situation. Examples of questions were: “*Can you please tell us from the morning to the evening how it is when your family member had the stroke?”* and *“How was your everyday life before your family member had the stroke? Can you describe the differences?”*

The interviews were conducted by one of the authors in the participants’ preferred language, Luganda or English, and they were audio-recorded. All the interviews were transcribed verbatim by a person that was fluent in both languages. After the interviews conducted in Luganda had been transcribed, they were translated into English.

### Assessment instruments

The *Caregiver Burden Scale (CBS)* was used to assess the subjective burden of the participating caregivers assisting their person that had had a stroke [22]. The 22 items deal with the caregiver’s health, feeling of psychological well-being, relations, social network, physical workload, and environmental aspects. Each question is rated from 1 to 4 (not at all, seldom, sometimes, often). The total scores range from 22 to 88, with the higher score indicating a greater burden. The scale has been shown to have good construct validity and test-retest stability [22, 23] and has previously been used in studies regarding caregivers to persons who have had a stroke [24].

The *Life Satisfaction checklist (LiSat-11)* is a generic, self-report checklist, assessing overall and domain specific life satisfaction in 11 questions [25]. The first question concerns global life satisfaction, and the remaining ten questions refer to satisfaction with their vocational situation, financial situation, leisure situation, contacts with friends and acquaintances, sexual life, ability to manage self-care, family life, partner relationship, physical health, and psychological health. The responses range along a six-grade ordinal scale from 1 (very dissatisfied) to 6 (very satisfied). The responses to the questions are dichotomized, and scores of 5–6 indicated “satisfied” and the scores 1–4 indicated “dissatisfied”. This is considered to be a valid scale reduction [25]. LiSat-11 and has been used in a number of studies with a sample of caregivers to people with stroke [11, 12, 26, 27].

Most of the data collection was conducted in the families’ home environments, while some was performed at the rehabilitation unit or in a room in the Makarere University area in Kampala. All places chosen for data collection were based on the families’ choice. During some of the interviews, more than one family member was present adding his/her views of the family’s life situation. In the Ugandan context, it is common to live together as an extended family and share the responsibility for the family as a whole. Therefore, it was natural to participate even in these family matters.

### Data analysis

Latent qualitative content analysis was used to analyze the interviews [28]. The analysis sought to identify subcategories and categories that appeared to be important in the experiences of the caregivers’ life situations. In the first step, the text was read through by two of the authors (GE, SG) in order to become familiar with and get a grasp of the content in the data. In the second step, the participants’ statements were divided into meaning units that were condensed further and related to each other through their content. The meaning units were then coded in a way that was close to how the participants had expressed themselves. To assure trustworthiness, the author (GE) discussed the coding and preliminary categories with the co-author (SG) until consensus was reached. In the last step, the codes were grouped into subcategories that were eventually summarized into five categories: *Feels obligated but is just a natural commitment*; *a tightly scheduled everyday life; being the supporting relative*; *the caregivers´ approach as rehabilitators*; and *being supported by the rehabilitation intervention.*

Descriptive statistics was used to analyze the information from the assessments.

## Results

The caregiver burden among the caregivers and their perceived life satisfaction are presented in Table 1, after which the caregivers’ experiences of their life situation are described.

**Table 1:** Self-reported levels of caregiver burden using Caregiver Burden Scale and life satisfaction using the LiSat-11 checklist (*n* = 12)

**Caregiver Burden Scale; median (range)**	**46.5 (28–82)**	
**Li-Sat-11; dichotomized**	**Items 1–11**	**Satisfying (5–6) /** **Dissatisfying (1–4)**
	Life as a whole is	4/8
	My vocational situation is	4/8
	My financial situation is	0/12
	My leisure situation is	2/10
	My contacts with friends and acquaintances are	5/7
	My sexual life is	7/5
	My ability to manage my self-care (dressing, hygiene, transfers, etc.) is	7/5
	My family life is	4/8
	My partnership relation is	5/7 – missing; n = 2
	My physical health is	6/6
	My psychological health is	7/5

The findings from the interviews with the caregivers are presented in the five categories that emerged in the analysis.

### Feels obligated but is just a natural commitment

Many of the relatives expressed an obligation to care for their daughter, mother, father or aunt when they had the stroke – it was just a natural commitment. It appears that they saw it as a commitment that they had to take on, self-imposed as a duty for their family member, as if being the mother obligated it. Being the caregiver could also be a demand from the rest of the family, as if being the first born, the first-born daughter, or the one among the children that earned the least, meant they were the ones chosen for this assignment. When the relatives told about their caregiving it was described as a very natural situation, something they wanted to do even if it was hard sometimes.

One caregiver said: *“It is not a sacrifice, he is my father, I can help him.”* Their everyday life had to change a lot for them to be able to fulfill this commitment, for example many had to quit their jobs and did not know if and when they could take up new employment. Others had to leave their home and families to move in with the family member with stroke. One participant left her children to live on their own to be a caregiver as her aunt needed her. She occasionally ran to visit her children a bit to advise them not to behave incorrectly and provide them with some food when possible. She expressed that *“they remain home on God’s mercy*.*”* Over the weekend, her aunt had someone else visiting and the niece was then able to be in her own home with her children.

The commitment of being a caregiver was extremely taxing and interfered a lot with their everyday lives. For some it meant that they could not even sleep at night. A daughter to a person with stroke expressed it thus: “We *really had a lot of struggling with her.”* Other family members with stroke did not need that much monitoring, but caregivers still felt an obligation to check on them when they were at work or had not been around for other reasons. Despite these big changes in their lives, it was not expressed as being a loss.

### A tightly scheduled everyday life

Daily life was busy for most of the caregivers. In addition to being a caregiver to their close family members, they also had their own family and children around and some worked part-time from home. Household chores often meant a heavy workload as most of the activities were performed by hand without home appliances like, for example, washing machines. Large families often lived together in small houses. To carry out the daily work for the whole family, the caregiver needed to plan carefully. One of the participants verbalized it thus: “I *had to program myself so everything fitted in the daily schedule.”*

The caregivers expressed how their days started early in the morning, at about 5 a.m. or even earlier, with assistance of self-care for their family member and exercises performed together before providing breakfast. The exercises varied but they all contained physical training, such as walking, arm and leg exercises, and some of the caregivers also massaged both arm and leg to *“make the weight go,”* meaning lessening the paralysis for the person who have had a stroke. One caregiver included dancing Zumba with his family member as a morning exercise as the person with stroke liked it very much and it made the whole body move.

The morning schedule before breakfast often also contained giving medication and assisting with taking a shower, which often meant warming and pouring water over the person with stroke. Lunch was provided for those that spent their midmornings at home. Some family members with stroke had appointments with a physiotherapist in the morning and they went to the rehabilitation clinic at 9 a.m. and the caregiver assisted with the exercises. The training often lasted the whole morning and they returned home at 1 p.m. For all persons with stroke, the afternoon included some rest at first and then the caregiver did some exercises together, as they had been urged to do by the physiotherapist or the OT.

The late afternoon included bringing afternoon tea and preparing supper. For some it also included pounding groundnuts to sell in the market, iron clothes, etc. In the evening, some of them needed to assist with preparing clothes and going to bed. One caregiver narrated her day thus: *“When I wake up, I wash the face, then I go there and pick milk as the patient [aunt] is asleep that she will use for the day, I boil the milk as I do other things like mopping, washing the dishes, sweeping the compound, preparing food. After I have finished, I get her out of bed quickly around 9.30 or 10 a.m. I give her also to wash her face, she does everything. Then I give her medicine and she swallows it. When we are done, she sits for a while. Thereafter I give her tea, she takes her tea and I go back to prepare lunch and bring it about 1.00 p.m. If there are some clothes to wash, I wash them. If it is a day for walking, we walk, we go round slowly, slowly with our stick. She can get out of the bedroom herself. I leave her to herself when I see she is not in pain. I leave her to do by herself, sometimes she bathes by herself at about 4 or 5 p.m. She comes and sits here; she does not sleep a lot.”*

Overall, being a caregiver to a family member who has had a stroke in Uganda resulted in a very tightly scheduled everyday life.

### Being the supporting relative

The caregivers reflected on the changes that the stroke entailed and described their sense of loss of the person their family members had been before they had the stroke. Some of them also shared the fear they had initially had that their family member would not recover. One caregiver narrated her eagerness to find a way to communicate with her mother and the frustration she felt at not being able to make sense of what she wanted. The importance of having a way to communicate was also described by other caregivers, and that it was depressing not to understand or know what the family member wished to do.

Some caregivers shared their experience of having to take care of their family members *“just like a baby we just turn to, she used diapers, we feed her*.*”* This time period with constant need of caregiving and the relief that the family member slowly, slowly improved and was able to sit and eat by him/herself and so on was something that the caregivers referred to. Some caregivers also expressed that at first they also felt sad for their sick family members, which was hard. Other caregivers felt mentally disturbed by having a family member that could not manage for him/herself.

One of the family members with stroke needed continuous surveillance by his caregiver, which was experienced as demanding. This family member needed assistance in changing his diapers and checking his blood sugar as he had serious diabetes. Another caregiver felt a lot of stress at not being able to leave her mother and go to work as it did not feel safe to go, which was a huge change in her life situation.

Some of the caregivers also expressed concern that their family members did not improve enough to be able to live in their homes in the village. Some were also concerned about the difficulties in getting access to, for example, physiotherapy when leaving the city and moving back to the village. On the other hand, one caregiver encouraged the family member to move back to the village as she felt much better being there. Even if she could not manage so much of the housework, in some ways she felt she was productive with the activities she managed to do. She could walk around and meet her neighbors in the village. This caregiver hired persons that could assist her with the activities she could not perform and thereby made it possible for the family member to stay in their own home. So, having a family member with a stroke could also mean financial support and taking care of a family member that was sick could be very expensive. The family needed to pay for medicine and transport, and they also had higher costs for food. One caregiver could only afford to cook one meal per day. Another caregiver felt stressed because she could not afford to provide her mother with what she wanted and needed. While living in the city, they had to buy vegetables at the market as they could not grow any themselves as they were used to doing in the village.

### The caregivers’ approach as rehabilitators

The caregivers’ tried to do the best they could to enhance their family members’ recovery and therefore made huge efforts. Many of them saw the family member taking part in the rehabilitation program as a very serious commitment. They felt responsible for the rehabilitation and doing whatever was expected of them to get the best out of the training they were supposed to assist in. One of the caregivers said that *“we do the exercise everyday all the time, we participate.”* She went on to express why this was so important: *“Yes, for us we want our mother to, want her, we want our mother to be back again.”*

All the caregivers appreciated the rehabilitation intervention F@ce that their family members had the opportunity to take part in. Their approaches regarding to what extent they should take on responsibility for the rehabilitation differed. One daughter was clear about the necessity for her mother to keep responsibilities in general and for parts in the rehabilitation that she could physically and mentally manage. For example, the family member with stroke handled contact with the OT and read the SMSs when she received them. She also answered the SMSs herself in the afternoon after doing the activities. Her daughter expressed that she was satisfied with not being involved and responsible for everything in the planning and execution of the different parts of the F@ce intervention as her mother took on this obligation. This family member was eager to take on as much housework as was manageable and the same daughter found ways to adapt chores and found simple activities that the family member could perform successfully and thereby feel useful again. This was also seen as a part of the rehabilitation even though they were not the specified activities *per se* that they had agreed on as goals for the rehabilitation.

Two caregivers whose family members got aphasia because of the stroke used different strategies to stimulate the talking. One, which family member was incontinent at first, agreed with her mother that she should communicate to her when she needed to go to the toilet and that induced the mother to start talking. The other daughter’s strategy was to play music with songs in the local language which the mother enjoyed and they started to sing together. After a while, this mother was able to say some words and answer questions.

Another caregiver felt like a counselor to her relative, trying to find activities or things that could benefit the family member’s recovery. For example, she hired a physiotherapist to come and provide training in the home to help the family member sit and stand. The same caregiver also took on an encouraging approach, by trying to make the family member improve in her daily activities. The caregiver thought of it as a successful approach but at the same time she also expressed that it was hard, and that the family member did not really bother about the suggestions and ideas brought up by the caregiver. She reflected that information and planning of the training worked better when it was delivered by someone who did not belong to the family, as in this case when the OT suggested activities and informed why they would be beneficial for the family member with stroke. The family member’s personality also influenced which kind of approach the caregiver chose to have. This same family member had not liked to take orders earlier in life and therefore it was not possible to force the family member to do the activities agreed upon. Therefore, the caregiver chose a playful way of reminding and assisting in the daily training. Sometimes it worked, sometimes not, but this was thought of as being the only way to succeed.

One caregiver narrated that they continued to work on the activities that were goals in the F@ce rehabilitation although the intervention had ended. Another caregiver reflected on how it was for her mother not being as self-sufficient as before and needing to practice how to dress herself again. She expressed her thoughts thus: *“Routines are very important in a person’s life. If somebody dresses, there are so many things which you can’t explain. There is thinking which one do I put on first, which one do I do then, as you do this the brain is thinking and is connecting with the arm and then you know. Almost the whole body is doing the exercise rather than somebody just coming and doing physiotherapy activities.”*

Being a caregiver to the family member who had had a stroke also meant that you became the person who would support them in resuming their activities. The caregiver became the one who would be responsible for the rehabilitation in the home, albeit with very little knowledge. The caregivers recounted that they were grateful that their family member had been allowed to participate in this new intervention as it meant that someone else also had a responsibility in the rehabilitation process and they received support in how to assist in the intervention.

### Being supported by the F@ce rehabilitation intervention

All caregivers shared their appreciation of the F@ce intervention, and all family members had improved in most of the activities they had as goals for the rehabilitation. They were satisfied that their family members were more independent in self-care, as for example one family member could take a shower on her own now which was important for her self-image *– “the intervention tries to give life back to her*.*”* Further the same family member with stroke also had found activities she was happy with, such as new ways of socializing with friends and neighbors, which was also very satisfying for her caregiver. Another caregiver expressed that contact with the OT was very important for his daughter: *“She feels like there is somebody who loves her, so it changes life.”*

Many caregivers expressed that they liked having contact with the OT. They also enjoyed having the OT and the researcher in their home at the start of the F@ce rehabilitation and at the follow up meeting. Other caregivers expressed their appreciation of their visit and the information and advice they provided in their home, explaining to the family member that they could help themselves to do things, that the muscles could refresh and they could go back to normal. It was after these types of conversations that the caregivers were inspired to encourage their family members to try to do activities themselves. The family members started to think about being independent, trying for example to take a bath on their own.

Several caregivers valued the OT’s telephone calls and the possibility to talk with them and that they cared about their family member and asked about how he/she was doing. One caregiver said, *“the calling used to wake me up”* and the SMSs were experienced as showing interest in the progress. Several caregivers also expressed that the calls from the OT were a relief since just knowing that these calls would come twice a week comforted them and decreased the stress they felt. The phone calls gave hope of a progress and the advice on how to assist the family members was appreciated – *“we really benefited because it gave us the free will in our hearts*.*”* They also had the chance to talk about their own situation and the fears they felt, which alleviated their worries.

## Discussion

The life situations had changed a lot for the twelve caregivers to the family members who have had a stroke in the urban area of Kampala. Half of them had given up their job or changed their work situation and had changed their living situation; moved from their own home to their family member’s home to manage the caring situation. Although their lives had changed substantially, they did not describe these alterations as being a big loss. Their everyday lives were busy, assistance and support to the family members were provided throughout the day together with performing household chores and assisting with training. The caregivers were concerned about their family members, whether they would recover enough to live on their own, as well as about the family’s financial situation. They felt responsible for assisting the family members in the intervention and appreciated the opportunity to participate in it, for the family members’ sake as well as for their own, and they felt supported by the intervention in their caregiving role.

There are several findings from the study that are of interest to discuss. The caregivers’ commitment to being caregivers despite great changes in their everyday life and their description of the burden of being caregivers will be discussed in relation to how they rated the caregiver burden and satisfaction with life on assessment instruments. The caregivers’ life situation in the context of being supported by an occupational therapist during a rehabilitation intervention will also be discussed below.

Most of the caregivers in this study were female, which is similar to previous studies [9, 10, 29] and three fourths of them were daughters to the persons with stroke. This reflects the Ugandan culture where the women are those having to take the responsibility for caring for family members even if they work fulltime [10]. All participants expressed that it was a natural commitment for them to take on the caregiver role, whether it was self-imposed or it was decided by the extended family that they were the most suitable person to take on the assignment. This agrees with the cultural pattern and values in Uganda that young people respect their older family members and thereby the norm is to provide care for those in the family that need it [30]. All participants in this study seemed to embrace these values, even if that implied having to quit their job and reduce the family income.

Even if it was natural to take on the caring of a family member, the caregivers nevertheless described being burdened by the demands the caregiving placed on them, which has been described before [10, 18]. They also rated their caregiver burden relatively high on the assessment, though similar to ratings by Swedish caregivers [11, 12], who rated on the same scale as those in this study. Perceived high caregiver burden has been seen before in African contexts [15, 29], with higher ratings than in samples in high-income countries. Other scales were used in the African studies, and one might consider that utilizing a scale developed for use in a Swedish context, and not culturally adapted, might have influenced the result. Therefore, the result would be interpreted with caution. Another consideration that one might reflect on regarding the perceived caregiver burden is that, in this study, the caregivers were provided with support in their caregiving role by the OT providing the F@ce intervention [20], which might have had a bearing on their perceived burden.

On the estimation of life satisfaction, two thirds of the sample rated their satisfaction with life as a whole as dissatisfying, which is a greater proportion than in Swedish studies using the same scale [12], even in groups of caregivers where their family members have been part of a rehabilitation intervention [11]. Further, all participants rated their financial situation as dissatisfying, which corresponds well with their narrated experiences of their everyday life and with previous findings [10]. Two thirds or more also found the vocational and leisure situation dissatisfying as well as their family life. These ratings also agree with the stories told in the interviews. Further, Akosile et al. [9] found that female gender was related to lower quality of life. One could consider comparing the results in the domains of self-reported life satisfaction with findings from other parts of the world, but as the life situation is so different and support from society to caregivers varies a lot between contexts, we will refrain from that. Furthermore, the LiSat-11 scale was developed in Sweden and is used a lot in rehabilitation samples all around the world, but seldom among caregivers and not in African contexts, whereby the results are difficult to compare to similar contexts.

The support the F@ce intervention brought to the families was highly appreciated as it improved the performance of valued/desired activities among the family members and thereby slightly decreased the pressure on the caregivers. The caregivers also felt supported by the information on stroke, how the consequences of stroke could be lessened by being active and how the caregivers could assist in the rehabilitation process. This information was provided to them during the home visits and in the regular telephone calls. Barbic et al. [31] identified that recognition of the changed life situation after stroke and support to caregivers from family, friends and healthcare was an important aspect to enable caregivers to cope better within their role. Caregivers in Uganda have requested information on stroke and basic skills required to handle a person that has had a stroke, follow-up after discharge from hospital, and active listening to the caregivers’ concerns [10]. Parts of that might have been provided to the caregivers in this study as a component of the intervention which might have strengthened them in their caregiving role. Unmet needs of caregivers to persons having had a stroke has, in a recent review, mainly including studies from Western countries, identified similar issues [32]. The review revealed unmet needs consisting of lack of information on stroke, rehabilitation, communication after stroke and how to provide support. Furthermore, the caregivers in the reviewed studies requested support including preparation for the caregiving and the home environment, support in the actual caregiving and rehabilitation process, support in sustaining living such as financial, health and family matters, and also emotional support to manage caregiving [32]. Evidently, the need for information and support from caregivers appears to be universal and needs to be offered to improve the stroke rehabilitation service and would preferably be targeted to meet the specific requirements in various contexts [32]. Meeting the families in their homes, as was the procedure within the F@ce intervention, might have informed the OT/researcher to be able to provide information and support deemed appropriate by the caregivers, as their contribution was so appreciated.

There are limitations to consider regarding the study. The sample is very small, albeit including the majority of those that were taking part in the F@ce intervention. Even if this small sample was representative regarding gender in the Ugandan context, these caregivers all lived in urban or suburban areas, which is the case of only a minority of people in Uganda. Further, the assessments used were not adapted to the cultural context and the ratings should therefore be interpreted with caution. Use of these assessments might however be looked upon as an approach to see whether the ratings harmonized with the stories told. It would however have been better to utilize assessment instruments previously used in the study context to be able to compare. Despite these limitations, this study provides unique information on the life situation for caregivers to family members who had had a stroke in Uganda and were provided with rehabilitation and support in their home environments. This knowledge can be a basis for the development of guidelines for support to caregivers that preferably will be implemented in health policies and care practices.

## Conclusions

The life situation changed substantially for those that took on the caregiver role when a family member had had a stroke, even if it was viewed as a natural commitment. Everyday life became challenging for them with an increased workload at home, caregiving responsibilities, giving up a job and a worsened financial situation. This situation burdened the caregivers and their satisfaction with life was low. The F@ce intervention was appreciated and entailed support also to the caregivers. They felt responsible for their family member’s participation in the rehabilitation and did whatever was required to enhance their family member’s recovery. The support provided included information on stroke and advice on training and caregiving, and regular contact with the occupational therapist was comforting and alleviated stress and worries. Access to support for caregivers in their demanding role is needed, something which has also been emphasized in earlier research.

## Data Availability

The datasets supporting the conclusions of this article are available at the Division of Occupational therapy, Department of Neurobiology Care Sciences and Society, Karolinska Institutet, Stockholm, Sweden. E-mail: susanne.guidetti@ki.se Part of the dataset supporting the conclusions of this article are included within the article and its additional files.

## Abbreviations

ADL: Activities of daily living
LiSat-11: Life Satisfaction checklist
QoL: Short message service
OT: Occupational therapist
CBS: Caregiver Burden Scale

## References

[1] Katan M, Luft A (2018). Global Burden of Stroke. Semin Neurol.

[2] Owolabi MO, Akarolo-Anthony S, Akinyemi R, Arnett D, Gebregziabher M, Jenkins C (2015). The burden of stroke in Africa: A glance at the present and a glimpse into the future. Cardiovasc J Afr.

[3] Owolabi M, Olowoyo P, Popoola F, Lackland D, Jenkins C, Arulogun O (2018). The epidemiology of stroke in Africa: a systematic review of existing methods and new approaches. J Clin Hypertens.

[4] Norrving B, Kissela B (2013). The global burden of stroke and need for a continuum of care. Neurology.

[5] Guwatudde D, Mutungi G, Wesonga R, Kajjura R, Kasule H, Muwonge J (2015). The epidemiology of hypertension in Uganda: Findings from the national non-communicable diseases risk factor survey. PLoS ONE.

[6] Kamwesiga JT, Eriksson G, von Koch L, Guidetti S (2018). The impact of stroke on people living in central Uganda: A descriptive study. Afr J Disabil.

[7] Masuku KP, Mophosho M, Tshabalala MD. ‘I felt pain. Deep pain… Experiences of primary caregivers of stroke survivors with aphasia in a South African township. Afr J Disabil. 2018. doi:10.4102/ajod.v7i0.368. 10.4102/ajod.v7i0.368 PMC5843930 29535917

[8] Saban KL, Hogan NS (2012). Female caregivers of stroke survivors: Coping and adapting to a life that once was. J Neurosci Nurs.

[9] Akosile CO, Okoye EC, Nwankwo MJ, Akosile CO, Mbada CE (2011). Quality of life and its correlates in caregivers of stroke survivors from a Nigerian population. Qual Life Res.

[10] Namale G, Kawuma R, Nalukenge W, Kamacooko O, Yperzeele L, Cras P (2019). Caring for a stroke patient: The burden and experiences of primary caregivers in Uganda – A qualitative study. Nurs Open.

[11] Bertilsson A-S, Eriksson G, Ekstam L, Tham K, Andersson M, von Koch L, Johansson U (2016). A cluster randomised controlled trial of a client-centred ADL intervention for people with stroke: one year follow up of caregivers. Clin Rehabil.

[12] Bergström A, Eriksson G, von Koch L, Tham K (2011). Combined life satisfaction of persons with stroke and their caregivers and the relation to the impact of stroke on everyday life. Health Qual Life Outcomes.

[13] Jaracz K, Grabowska-Fudala B, Gorna K, Jaracz J, Moczko J, Kozubski W (2015). Burden in caregivers of long-term stroke survivors: prevalence and determinants at 6 months and 5 years after stroke. Patient Educ Couns.

[14] Mcpherson CJ, Wilson KG, Chyurlia L, Leclerc C (2011). The caregiving relationship and quality of life among partners of stroke survivors: A cross- sectional study. Health Qual Life Outcomes.

[15] Akosile CO, Okoye EC, Adegoke BOA, Mbada CE, Maruf FA, Okeke IA (2013). Burden, health and quality of life of Nigerian stroke caregivers. Health Care Current Reviews.

[16] Akosile C, Banjo TO, Chiebuka Okoye E, Olanrewaju Ibikunle P, Odole AC (2018). Informal caregiving burden and perceived social support in an acute stroke care facility. Health Qual Life Outcomes.

[17] Vincent-Onabajo G, Ali A, Hamzat T (2013). Quality of life of Nigerian informal caregivers of community-dwelling stroke survivors. Scand J Caring Sci.

[18] Kalavina R, Chisat E, Mlenzana N, Wazakili M (2019). The challenges and experiences of stroke patients and their spouses in Blantyre, Malawi. Malawi Med J.

[19] Greenwood N, Mackenzie A, Cloud GC, Wilson N (2009). Informal primary carers of stroke survivors living at home–challenges, satisfactions and coping: A systematic review of qualitative studies. Disabil Rehabil.

[20] Kamwesiga JT, Eriksson G, Tham K, Fors U, Ndiwalana A, von Koch L, Guidetti S (2018). A feasibility study of a mobile phone supported family-centred ADL intervention, F@ce™, after stroke in Uganda. Global Health.

[21] Creswell JW, Plano Clark VL. Designing and conducting mixed methods research. 3rd ed. Los Angeles: SAGE; 2011.

[22] Elmstahl S, Malmberg B, Annerstedt L (1996). Caregiver’s burden of patients 3 years after stroke assessed by a novel caregiver burden scale. Arch Phys Med Rehabil.

[23] Visser-Meily A, Post M, Riphagen I, Lindeman E (2004). Measures used to assess burden among caregivers of stroke patients: a review. Clin Rehabil.

[24] Björkdahl A, Nilsson AL, Sunnerhagen KS (2007). Can rehabilitation in the home setting reduce the burden of care for the next-of-kin of stroke victims?. J Rehabil Med.

[25] Fugl-Meyer AR, Melin R, Fugl-Meyer KS (2002). Life satisfaction in 18- to 64-year-old Swedes: in relation to gender, age, partner and immigrant status. J Rehabil Med.

[26] Hedman A, Eriksson G, von Koch L, Guidetti S (2019). Five-year follow-up of a cluster-randomized controlled trial of a client-centred activities of daily living intervention for people with stroke. Clin Rehabil.

[27] Forsberg-Wärleby G, Möller A, Blomstrand C (2004). Life satisfaction in spouses of patients with stroke during the first year after stroke. J Rehabil Med.

[28] Graneheim UH, Lundman B (2004). Qualitative content analysis in nursing research: concepts, procedures and measures to achieve trustworthiness. Nurse Educ Today.

[29] Okeke PC, Oparah SK, Oboke SO, Inem V (2020). Care Burden Correlates with Depression among Informal Caregivers of Stroke Patients: A Cross Sectional Study in Lagos, Nigeria. Int J Caring Sci.

[30] Blunt P, Jones ML (1992). Managing organisations in Africa.

[31] Barbic S, Mayo N, White C, Bartlett S (2014). Emotional vitality in family caregivers: content validation of a theoretical framework. Qual Life Res.

[32] Zawawi NSM, Aziz NA, Fisher R, Ahmad K, Walker MF (2020). The unmet needs of stroke survivors and stroke caregivers: A systematic review. J Stroke Cerebrovas Dis.

